# Comparison of accepted and unaccepted living kidney donors: one-center experience

**DOI:** 10.1080/0886022X.2018.1450758

**Published:** 2018-03-26

**Authors:** Aleksandra Kezić, Svetlana Kovačević, Jelena Marinković, Stojanka Ristić, Dragana Radivojević, Radmila Blagojević-Lazić, Ljubica Djukanovic, Visnja D. Ležaić

**Affiliations:** a School of Medicine, University of Belgrade, Belgrade, Serbia;; b Department of Nephrology, Clinical Centre of Serbia, Belgrade, Serbia

**Keywords:** Kidney transplantation, living donor, donor evaluation, selection criteria, donor risk

## Abstract

**Background:** Kidney transplantation from living donors (LD) has stagnated in many countries. This study aimed to check whether correction of LD selection practice could increase the number of kidney transplantations.

**Methods:** From January 2003 to December 2012, 241 potential adult LD were evaluated in our hospital. Outcome (mortality and end-stage renal disease-ESRD) of accepted LD (182) was compared with unaccepted (59) donors.

**Results:** Mortality of LD was comparable with that for the standardized Serbian population (SMR = 1.104; 95% CI (0.730–1.606). Among evaluated potential LD, almost every fourth had been unaccepted, but reasons were modifiable in 42.4% of them. In pre-donation period unaccepted donors were significantly older, measured glomerular filtration rate was lower, with higher 15-year and lifelong projected ESRD risks than accepted donors. Despite this, ten years outcome of both groups LD was similar: none of LD developed ESRD, 9.8% of accepted and 11.8% of unaccepted LD died (*p* = .803).

**Conclusions:** During an average of 101 months of follow-up mortality of accepted LD did not differ significantly as compared to the age standardized Serbian population and none of them developed ESRD. In examination of potential LD, the use of accurate and precise methods for kidney function estimation and the evaluation of risk for ESRD and mortality as well as treatment of modifiable contraindications for kidney donation are necessary.

## Introduction

Living kidney donor (LD) transplantation is the optimal therapy for many patients with end-stage renal disease (ESRD), providing numerous clinical benefits when compared with prolonged dialysis or deceased donor kidney transplantation. These include better patient and graft survival and improved quality of life [1,2]. Although LD transplantation has recently stagnated in many countries in Europe [3], as well as in the USA, Canada, Australia, New Zealand and Brazil, it has continued to grow substantially in some other countries, such as Japan and South Korea [4]. The declines in LD seem to be temporally associated with many causes some of which are: economic recession, attention to the financial risks of kidney donation for individuals with few savings or little income, an aging donor pool, reduction in family size and changes in donor selection criteria [5]. In addition, some authors claimed poor long-term prognosis for LD with evidence of higher risks for developing ESRD or mortality [6–8]. In many of the observational studies, donor outcomes were compared with general population controls, which could mask any increased risk related to donation. Also, LD undergoes extensive medical and psychosocial assessment and therefore they are healthier than the general population. All this underlines the importance of donor comparisons with adequate controls. In order to help evaluation and selection of LD candidates, Grams et al. [9] recently proposed a kidney failure risk projection model that provides estimation of a kidney donor candidate’s chance of developing ESRD over a 15-year period using population-based data.

Living kidney donation started at our Clinic in the early 1980s. Since then, the proportion of such donations increased up to 100% in the early 1990s and thereafter declined to 15.2% in 2010. In approximately the same period the relative number of kidney transplantations from deceased donors rose. According to the last annual report on RRT in Serbia for 2014, 843 patients started dialysis (108.3 pmp), while kidney transplantation was done in nine patients (1.3 pmp), indicating that kidney transplantation does not meet the needs in Serbia [10,11]. In the circumstances, with so few kidney transplantations the question arose whether the rejection of LD was always justified. Therefore, an analysis of the causes of rejection of LD as well as the outcome of potential unaccepted LD seemed to be of particular interest. In the present retrospective study we compared the outcome of LD accepted and unaccepted for kidney donation with the aim to check whether it could be possible to increase the number of kidney transplantations by correction of the selection practice.

## Materials and methods

From January 2003 to December 2012, 241 potential adult LD were evaluated in our hospital. Among them, 182 donated a kidney. Their pre-transplantation results were compared with potential unaccepted LD. Kidney donors were regularly monitored for 6 to 12 months after nephrectomy and annually thereafter. Follow-up data on death or ESRD obtained either from donor files or by telephone communication with donor or family member, was available to March 2016 for all examined donors, both for accepted and unaccepted.

All potential LD were evaluated according to our guidelines after giving informed consent for the pre-transplant examination and final informed consent for kidney donation. The Ethics Committee of the Clinical Center of Serbia evaluated and approved those who gave a kidney. Besides ABO and HLA matching with the recipient, the evaluation of potential LD included careful history, physical examination, and tests to detect conditions that may preclude safe donation: uncontrolled hypertension (with/without antihypertensive medications), cardiovascular disease (ischemic heart disease, cardiac failure), diabetes, obesity [body mass index (BMI) > 30 kg/m^2^], long-term use of nonsteroidal anti-inflammatory drugs, smoking habit, alcohol abuse and a family history indicating an increased risk of renal disease. In addition, the following conditions were considered: routine cancer screening, screening for transmittable infections and anatomic evaluation focusing on the vascular supply and structural integrity of the kidneys. The assessment also included psychosocial screening, for motivation, voluntary consent and mental suitability. According to our guidelines, only relatives with the same or a compatible ABO blood group were evaluated as potential donors, with a minimum of haplotype matching and negative cross-match reaction. In addition, only HBsAg and anti-HCV negative persons, and those without diabetes (fasting glucose <6.1 mmol/L and HbA1c < 6.5%) [12] were accepted as kidney donors.

Kidney function was assessed by measurement of 24 h urinary albumin and protein excretion, urinary sediment analysis, determination of creatinine clearance and glomerular filtration rate measured with technecium ^99m^DTPA (mGFR) [13]. The variation coefficient of this method in our institution was 14.8%. In addition, measurement of serum cystatin C and albuminuria were introduced in the standard procedure of potential donor evaluation in 2008. Thus these parameters were not available for all evaluated donors. The minimum standards for donor acceptance were creatinine clearance >80 mL/min/1.73 m^2^, mGFR >80 mL/min/1.73 m^2^, cystatin C < 1.0 mg/L, albuminuria <30 mg/24 h and proteinuria <200 mg/24 h.

### Study variables

Demographic data (age, gender), habits (smoking, alcohol abuse), BMI and data on the presence of chronic ailments such as: cardiovascular diseases, hypertension, use of antihypertensive medications, pulmonary, gastrointestinal, genito-urinary tract and inflammatory diseases were included in the data base. Blood pressure values, data obtained by physical examination, kidney function and a structure evaluation were analyzed, too.

To assess the 15-year and lifetime risks of ESRD for kidney donor candidates, we used the online tool developed recently by Grams and coworkers [www.transplantmodels.com/esrdrisk^9^]. It provides an estimate of the 15-year and lifetime incidence of ESRD from a set of demographic and baseline (pre-donation) health characteristics including: age, sex, BMI, race, smoking history, systolic blood pressure, antihypertensive medications, urinary albumin to creatinine ratio, GFR estimated by Epidemiology Collaboration equation [14] and diabetes. It does not take into account any added risk a donor might incur due to the nephrectomy or resultant single kidney status.

### Statistical analysis

Continuous variables were tested for normal distribution using the Kolmogorov–Smirnov test. Normally distributed continuous variables are presented as mean ± standard error, while continuous variables that did not show a normal distribution are given as the median value and interquartile range (IQR). Categorical variables are reported as counts with percentages and were compared with the Chi square or Fisher’s exact test. The unpaired Student’s t-test was used to analyze differences in continuous variables divided into two independent groups. Univariate and multivariate Cox regression analyzes were used to determine the association of the various predictors with time to all-cause mortality. Donor survival was calculated using Kaplan–Meyer analysis, and the comparison of survival curves was calculated by log rank test. Standardized mortality ratios (SMRs) for evaluated donors were calculated using data on deaths and age structure of the general population of Serbia [15]. SMR was calculated by dividing the observed number by the expected number of deaths. Statistical analyzes were performed using the statistical package for social sciences, version 17 (SPSS, Chicago, IL). Statistical significance was defined as *p* < .05.

## Results

**Figure 1.**
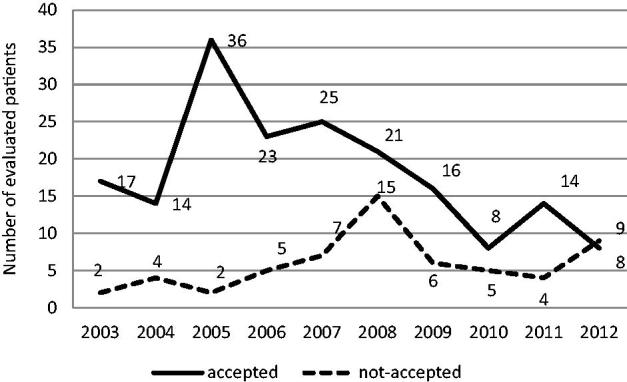
Number of potential living donors (accepted and unaccepted for donation the kidney) evaluated in the period from 2003 to 2012. The number of potential donors evaluated for donation diminished in the 10 years analyzed, especially after 2010. The rate of acceptance of patients decreased over the 10-year period, i.e., 95.8% of the evaluated donors were accepted in 2005 but 30.4% in 2010.

The number of potential donors evaluated for donation diminished in the 10 years analyzed, especially after 2010 (Figure 1). The rate of acceptance of patients also decreased over the ten year period, i.e., 95.8% of the evaluated donors were accepted in 2005 but 30.4% in 2010. The number of living kidney donations peaked at 36 in 2005 and declined by 87% to 8 in 2010. Among the evaluated LD, 177 (73.4%) were older than 50 years, and 33.3% of them were aged over 65 years.

The donor group did not have significantly different mortality compared to the age standardized Serbian population (SMR = 1.104; 95% CI (0.730–1.606) (Table 1). Taking into account donor age, the data showed that the donors older than 65 years were more likely to die. During the entire observation period 25 of potential donors died of which 14 were over 65 years old (Table 1).

**Table 1.** Death numbers and standardized mortality ratio (SMR) of evaluated donors.

Age	Person-years	Deaths
20–39	57.83	0
40–44	133.00	0
45–49	226.50	3
50–54	249.17	4
55–64	763.67	4
65–74	464.17	10
+75	93.92	4
Total	1988.25	25
Death rate	0.01257	
SMR (95% CI)^a^	1.104 (0.730–1.606)	

a According to the population in Serbia matched by age.

After complete evaluation 59 potential donors (24.5%) did not donate a kidney to their relatives. Reasons for not accepting evaluated donors were dependent on recipient and donor factors as presented in Table 2. Among factors dependent on the recipients those identified as other were discovered during examination and included: high risk of bleeding due to antiphospholipid syndrome (four patients) and hereditary macrothrombocytopaenia.

**Table 2.** Reasons of not accepting evaluated donors for donation.

	Reasons	Number of patients
Recipients	Deceased kidney transplantation	1
	Histocompatibility mismatches	4
	Cancer de novo	1
	Other^a^	5
Donors	Kidney disease^b^	5
	eGFR <80 ml/min/1.73 m^2^	5
	Kidney stone	2
	Multiple cysts	3
	Cardiovascular diseases^c^	3
	Needs prior surgery	2
	Cancer de novo (breast, lung)	2
	Older age	5
	Withdrawn before evaluation complete	21

a High risk of bleeding due to antiphospholipid syndrome or hereditary macrothrombocytopaenia.

b Albuminuria <30 mg/24 h, or proteinuria <200 mg/24 h, or persistent microhaemathuria.

c Uncontrolled hypertension (with >2 anti hypertensive medications), cardiovascular disease (ischemic heart disease, cardiac failure).

To answer the question whether we had made a good selection of kidney donors, unaccepted donors were compared with the accepted ones. The baseline characteristics of potential donors from each group are summarized in Table 3. At the beginning of evaluation, co-morbidities were similar in both groups. Unaccepted donors were older than the accepted ones due to the larger number of subjects older than 70 years. However, 37 (20%) of accepted donors were over 65 years of age, most of them providing kidneys up to 2009 (data not presented). Glomerular filtration rate measured with ^99m^DTPA was lower in unaccepted than in accepted donors group. Both 15-year and lifetime observed risks for ESRD were significantly higher for unaccepted donors than for accepted donors. Considering individual results for basal analysis, it can be noticed that two unaccepted donors had the highest lifetime risk of ESRD, due to proteinuria above 1 g/24 h plus persistent microhaematuria.

**Table 3.** Baseline data on studied potential donors at the time of pre-donation evaluation.

	Unaccepted donors *n* = 59	Accepted donors *n* = 182	*p*
Sex, f/m	24/35	65/117	.535
Age^a^, years	61.41 ± 1.13	56.76 ± 0.78	.0024
Distribution^b^			
20–39	1	9	.458
40–44	1	15	.128
45–49	6	24	.653
50–54	7	22	1.000
55–64	22	67	1.000
65–74	14	33	.349
+75	8	4	.002
Co-morbidities^b^			
Cardiovascular diseases	8 (13.6%)	20 (11%)	.641
Hypertension	24 (40.7%)	60 (30%)	.345
Pulmonary	3 (5.1%)	16(8.8%)	.577
GID	13 (22%)	22 (12%)	.086
GUT	9 (15.3%)	37 (20.3%)	.450
Inflammation	8 (18.6%)	11 (6%)	.091
Blood pressure, mmHg			
Systolic	119.52 ± 6.42	115.6 ± 4.13	.629
Diastolic	73.3 ± 3.84	72.0 ± 2.56	.795
^99m^DTPA mGFR, ml/min/1.73m^2^	78.47 ± 5.60	87.17 ± 3.79	.030
Habits^b^			
Smoking	10 (5.9%)	2 (13.2%)	.519
Alcohol abuse	1 (0.01%)	4 (2.2%)	1.000
Follow-up^c^, months	96	108	
IQR	60	48	
Events			
ESRD	0	0	.803
Death	7 (11.8%)	18 (9.8%)	
15 years projected ESRD risk	0.288 ± 0.048	0.194 ± 0.015	.013
Lifetime projected ESRD risk	0.669 ± 0.098	0.427 ± 0.03	.002
15 years projected ESRD risk, %			
<1	93.4%	98.4%	
1–2	5%	1.6%	
2–3 Lifetime projected ESRD risk, %	1.6%		
<1	81.4%	91.3%	
1–2	11.8%	8.7%	
2–3	3.4%		
3–5	3.4%		
Causes of death,^b^			
Cardiovascular diseases			
Malignancies	1	10	
Cirrhosis	1	2	
Unknown	5	6	

ESRD: end-stage renal disease; GID: gastrointestinal diseases; GUT: genito-urinary tract diseases; IQR: interquartile range; mGFR: measured glomerular filtration rate.

^a^mean ± SE, ^b^number of patients, ^c^median.

**Figure 2.**
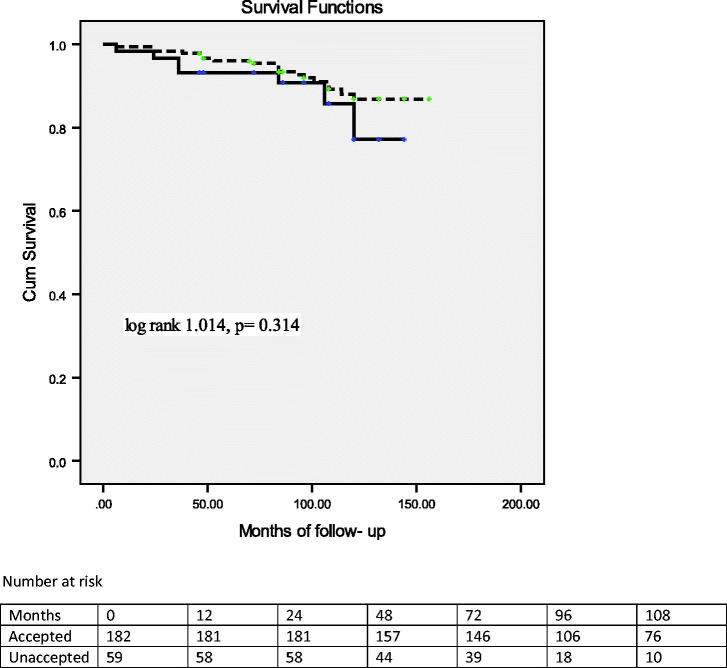
Kaplan–Meier curves of cumulative proportion surviving across those groups with number of donors at risk. Survival was not significantly different in the accepted donors full line (median 108 months; IQR 48) [log rank test 1.014, *p* = .314] when compared with the unaccepted donor group (median 96 months, IQR 60).

The median follow-up of all examined persons was 101 months (IQR 36). None of both accepted and unaccepted LD developed ESRD during the follow-up period. No significant difference was found between survival of the accepted LD (median 108 months; IQR 48) [log rank test 1.014, *p* = .314] as compared to the unaccepted LD (median 96 months, IQR 60). Kaplan–Meier curves of cumulative proportion surviving across those groups presented in Figure 2 showed no difference. During the observed period, there were 18 deaths among 182 accepted LD (9.8%), due to cardiovascular disease (10 donors), malignancies (one donor each: lung and breast cancer), and unknown causes in the remaining six donors. None of the donors died during or immediately after the surgical procedure. Among the unaccepted LD there were seven (11.8%) deaths, due to decompensate cirrhosis and prostate cancer (one subject each) and unknown causes in five of them (Table 3). The mean age of accepted LD at the time of death was 72 ± 10.7 years and death occurred 84 months (median value, minimum 6 months, maximum 120 months) after donation. The mean age of unaccepted LD at the time of death was 69.8 ± 9.0 years and death occurred 36 months (median value, minimum 6 months, maximum 106 months) after rejection (data not presented).

In order to determine predictors of death for all examined LD, all variables listed in the Methods section were included in the univariate Cox regression analysis. Since no variable has reached statistical significance but patients’ age, multivariable model has been tested with two most important variables i.e., donor’s age and group. Finally, donor age was selected as the only significant predictor of donor death (*p* = .007; exp B 1.067, 95% CI 1.018–1.118) indicating that an increase in age by 1 year points to a 6.7% increase in the risk of death, which is valid for the age interval examined here.

## Discussion

During the 10-year period 241 potential adult LD were evaluated in our hospital. Out of them almost every fourth one (59–24.5%) was not accepted for kidney donation and in 42.4% of them the reason for non-acceptance was modifiable: kidney stone, needs for surgery, unsatisfactory control of hypertension, but also better donor motivation. Comparison of unaccepted and accepted LD showed that in the pre-donation period the unaccepted LD were significantly older, their mGFR were lower and had higher 15-year and lifetime projected ESRD risk calculated by tool proposed by Grams and coworkers [9]. Despite to these differences, the long-term outcome of LD from both groups was similar. None of all examined LD developed ESRD and no difference was found in mortality between accepted and unaccepted LD as well as between all potential LD and healthy non-donors from general population.

Prior studies that compared survival of LD and healthy-matched non-donors reported inconsistent results. Ibrahim and coworkers [16] analyzed a cohort of 3698 donors and reported that survival of kidney donors, at a mean of 12.2 ± 9.2 years after donation, was similar to that of controls from general population life table estimates in the Human Mortality Database. They were matched for age, sex and race or ethnic group. A favorable outcome for kidney donors may be maintained more than 30 years after donor-nephrectomy [17,18]. Even more, mortality among LD aged over 70 years was not higher than healthy matched controls drawn from the NHANES-III cohort [19]. In fact, mortality was lower, probably reflecting higher selectivity among older LD than could be captured in the National Health and Nutrition Examination Survey III (NHANES-III; HR 0.37, 95% CI 0.21–0.65, *p <* .001) [16]. In contrast, Mjoen and colleagues [7] reported an increased risk of death in 1901 Norwegian kidney donors compared with healthy matched individuals from a regional population survey with a median follow-up of 15.1 years. The authors showed that during the first 15 years of follow-up, the difference in mortality risk between LD and healthy non-donors was 0%, but after 25 years the risk was 5%, indicating that every twentieth living donation was followed by a death associated with the nephrectomy up to 25 years afterwards (adjusted hazard ratio 1.30). Besides mortality, several recent studies showed that ESRD rates were almost 10 times higher among donors compared with non-donors [7,8]. Although several shortcomings were identified in the methodology in above mentioned studies [20], these alarming results as well as completely opposite results obtained by other authors indicated the need to monitor donor acceptance patterns and long-term donor outcomes.

While in previous studies either donors’ outcome or reasons for rejection of evaluated potential LD were examined, we accessed to this problem with a somewhat different purpose. In the analysis of 241 potential kidney LD, out of which 59 were not accepted for kidney donation, we analyzed not only the reasons for rejection for kidney donation but also compared the outcome of both accepted and unaccepted LD to verify whether the decision not to accept a group of LD was correct. The main purpose of the study was to check whether it could be possible to increase the number of kidney transplantations by correcting the selection criteria. After careful evaluation, 11 LD were not accepted due to the recipients’ reasons, mainly immunological mismatches and clinical contraindications. Among LD rejected due to donors’ reasons, two had disease requiring surgery pre donations (cholecystectomy in both), and two kidney stones, and these problems could be solved before donation. In addition, 21 LDs withdrew from donating a kidney mostly due to too long evaluation. The testing process is often very time-consuming, as it is a long wait for individual examinations, and LDs do not have priority in scheduling certain examinations compared to other patients. All this indicates that probably some of our rejected LD could proceed to live kidney donation. If our decision making process about the suitability of LD had been more efficient, we would probably have been able to increase the pool of LD in the analyzed period. In order to solve this problem, we changed the process of preparing potential donors and shortened the testing process, and we are always trying not to reject potential LD due to the modifiable reason. Romagnoli et al. [21] came to similar conclusion and proposed a dedicated diagnostic pathway allowing candidates to complete their evaluation in a shorter time that may improve the yield of donors.

Comparison of our evaluated potential LD showed that unaccepted subjects were older than accepted ones and donor age was the only significant difference between accepted and unaccepted potential LD. As age was selected as a predictor of donor death by Cox regression analysis it would seem that it was justified not accept older donors. Previously published data on the influence of donor age on the outcome and kidney transplantation results are inconsistent. Thus, Segev et al. [22] identified higher relative mortality among older than among younger donors. Namely, those aged 50–59 years (hazard ratio (HR) 3.3; 95% confidence interval (CI) 2.6–4.1) or 60 years or older (HR 9.4; 95% CI 7.3–12.1) were associated with a greater 12-year mortality rate when compared with donors aged 18–39 years (HR 1) in a sample of 80 347 live kidney donors. It is expected that diminished filtration function at baseline in older donors might impair the ability of the remnant kidney to perform adaptive hyperfiltration. This would promote progressive kidney disease or co-morbidities such as cardiovascular diseases. Having in mind our previous study, which showed that kidneys from older LD provide a statistically poorer transplant outcome [23], we have tried to be more strict in the selection and avoid LD older than 65 years. Consequently, a declining trend in the number of accepted older LD is observed since 2009. However, recent analyzes have given different arguments. Improvements in clinical transplantation procedures have produced excellent results from older LD, nullifying the effect of donor age on patient and graft survival. Therefore, donor age alone need not be an exclusion criterion for living kidney donation [24–26]. Data on the outcome of our potential LD are in favor of this. Despite Cox regression analysis selected donor age as a significant predictor of death, the percentage of deaths during the 10-year period was similar in both groups of LD, although unaccepted donors were significantly older than accepted ones. In addition, when considering age as a criterion for donation, it should be noted that older LD have fewer expected years of survival than younger LD. Older LD will experience a briefer period with a single kidney, which should reduce the opportunity for adverse consequences of nephrectomy [27]. Younger LD have the potential to develop and be exposed to renal and cardiovascular risk factors for a longer period of time than older LD, resulting in an increased lifetime risk of developing ESKD or premature mortality [28].

The assessment of renal function before donation is very important but our ability to accurately assess GFR is insufficient. We cannot measure GFR with iothalamate, iohexol, but with less precise DTPA and the introduce of more precise methods of measuring GFR in our LD selection process is mandatory. Nevertheless, during the monitoring period, none of our examined LD developed ESRD. This was due either to proper selection of potential donors or insufficient follow-up time afterwards. Previous studies suggest that renal risks take more than 10–30 years to become apparent [29,30] if they occur at all. Using the on-line tool of Grams et al. [9] we showed that unaccepted LD had higher average 15-year and lifelong risks for ESRD than accepted LD. The proposal of this ESRD risk projection model has provoked numerous comments but both the authors and those declaring certain remarks felt that it was necessary to estimate the model. Our study could not be considered as the right estimation of the model because it lasted for 10 rather than 15 years and it was used to predict ESRD risk in accepted and unaccepted LD. Nevertheless, it does not support the accuracy of the model, because during 10 years none of potential LD, both those with high and those with low risk for ESRD, developed ESRD. The authors of ESRD risk projection model themselves considered that although their tool should help clinicians in prediction long-term risk of ESRD for potential LD in predonation period, it could not replace comprehensive donor evaluation by an experienced team of doctors.

A key strength of the present study is its contribution to the justification of kidney transplants from LD in our developing country, in which the program for kidney transplantation from deceased donors is insufficient to meet the needs of our patients. In addition, it indicated that a lower threshold for acceptance of older LD could be reasonable, as well as that donor acceptance practice should be modify on a case-by-case basis. To this conclusion, we came by following up and analyzing the outcome of both groups of donors, i.e., accepted and unaccepted.

Several study limitations should be taken into consideration. Firstly, the number of evaluated LD was too small, especially in the unaccepted group, to perform analyzes on excess overall risk of LD. Thus, a larger database should be generated to conduct these analyzes. Secondly, follow-up of the LD was limited to five to ten years so we were not able to examine the impact of donor nephrectomy on long-term donor outcome i.e., mortality and ESRD of the remnant kidney due to the low incidence of these events. However, the strict outcome measures of death or ESRD do not really off insight into the future morbidity should these unaccepted donors have actually donated their kidneys. Finally, prospective studies with a longer follow-up period investigating the influence of medical factors on donor outcome could add important insights to this area.

## Conclusions

Our results showed that during an average of 101 months of follow-up mortality of accepted LD did not differ significantly as compared to the age standardized Serbian population. None of all examined LD, both accepted and unaccepted ones, developed ESRD, and no difference was found in mortality between these two groups. In examination of potential LD, the use of accurate and precise methods for kidney function estimation and the evaluation of risk for ESRD and mortality are mandatory. In addition, treatment of modifiable contraindications for kidney donation and faster decision making process on the suitability of donors are necessary.
